# Defining and Evaluating the Impact of Bleeding Severity on Time to Endoscopy and Mortality Risk: A Prospective Multicenter Cohort Study

**DOI:** 10.3390/jcm14051643

**Published:** 2025-02-28

**Authors:** Clelia Marmo, Cristina Bucci, Marco Soncini, Maria Elena Riccioni, Riccardo Marmo, on behalf of the GISED Study Group

**Affiliations:** 1 CEMAD Centro Malattie Dell’Apparato Digerente, Fondazione Policlinico Universitario Agostino Gemelli, Università Cattolica del Sacro Cuore, Largo Agostino Gemelli, 00168 Rome, Italy; 2 Gastroenterology and Hepatology Unit, AORN Santobono-Pausilipon, 80122 Naples, Italy; 3 Department of Internal Medicine, “A. Manzoni” Hospital, 23900 Lecco, Italy; marco.soncini1@gmail.com; 4 Digestive Endoscopy Unit, Fondazione Policlinico Universitario Agostino Gemelli, Università Cattolica del Sacro Cuore, Largo Agostino Gemelli, 00168 Rome, Italy; 5 Gastroenterology and Endoscopy Unit, “L. Curto” Hospital, ASL Salerno, 84035 Polla, Italy

**Keywords:** acute upper gastrointestinal bleeding, time to endoscopy, mortality, bleeding severity

## Abstract

**Background:** Upper gastrointestinal bleeding severity (BleSev) is commonly defined by evaluating different factors that are frequently interdependent on each other, expressing the same underlying cause. **Aim:** This study aimed to define the severity of a bleeding event and verify its impact on death risk and the time to endoscopy. **Methods:** We analyzed 12 factors (demographic, hemodynamic, biochemical, and clinical) that could be associated with BleSev. We identified the independent weight of each factor in predicting a composite endpoint (need for surgery, interventional radiology, and death) and the effect of the interactions between time to endoscopy and BleSev on death risk. **Results:** Clinical data of 2.525 patients were included. Of the 12 factors, 5 were retained in the final model as follows: altered mental status, systolic blood pressure ≤ 100 mmHg, blood urea nitrogen level ≥ 130 mg/dL, hematemesis, and hemoglobin level ≤ 8 g/dL (AUC performance curve, 0.79). We identified the following three classes of BleSev: low (0–1 points, 2.4%), intermediate (3–4 points, 8.6%), and high (≥5 points, 21.1%). When no factors were present, the death risk was 1%; when all factors were present, the risk was 45.5%. Notably, the death risk increased with BleSev but was generally independent of time to endoscopy. However, in high-risk cases, early endoscopy (within 6–12 h) was associated with a reduced mortality rate. **Conclusions:** This study defines a risk model for BleSev and highlights the need for targeted endoscopic timing strategies based on BleSev for optimizing survival rates. Patients in the highest risk category may benefit from more urgent endoscopic interventions.

## 1. Introduction

Gastrointestinal bleeding is a common cause of death worldwide, and the upper gastrointestinal tract is the most frequent site. Previous studies have indicated that the incidence and epidemiology of hospital admissions have been reduced to 61–78 cases per 100,000 individuals, and the causes of bleeding have changed over time [1,2,3]. Currently, the most frequent causes of gastrointestinal bleeding are nonvariceal, including peptic ulcers, vascular lesions, and malignancies, with esophagogastric varices being less frequent than previously reported. For upper gastrointestinal bleeding, the mortality rate varies from 2% to 10% [4].

Typically, patients with severe acute gastrointestinal bleeding present with hematemesis or melena and could be hemodynamically unstable, requiring prompt resuscitation. Therefore, bleeding event severity and underlying comorbidities are the most significant risk factors for mortality. Previous studies reported that bleeding severity (BleSev) is scored by evaluating presenting symptoms (hemodynamic shock, hematemesis, and melena), altered blood test results (hemoglobin [Hb] and blood urea nitrogen concentrations and coagulopathy), source of bleeding, and type of endoscopic findings [5,6,7,8]. However, these aspects are frequently interrelated, expressing the same underlying cause, rather than being distinct co-factors; therefore, the same factor may be weighted more than once in the overall severity estimation.

The aim of the study is to define BleSev and verify the impact of BleSev on mortality risk and time to endoscopy.

## 2. Materials and Methods

This was a prospective nationwide cohort study conducted from 1 January 2014 to 31 December 2015 in 50 hospitals in Italy from the Gruppo Italiano Studio Emorragia Digestiva (GISED) Study Group. Centers involved in the research were Public Hospitals, Private Hospitals, and University Hospitals.

All consecutive patients with ongoing overt acute upper gastrointestinal bleeding (AUGIB) were included and underwent endoscopy to confirm the bleeding source and causes.

Inclusion criteria were adult patients with acute upper gastrointestinal bleeding experiencing hematemesis/coffee-ground vomiting, melena (black tarry stool), or a combination of both, confirmed by upper endoscopy.

The following were the exclusion criteria: low or intermediate sources of gastrointestinal bleeding and unavailability/unwillingness to provide informed consent for data collection. Patients’ clinical characteristics, basic laboratory tests, clinical evolution during hospitalization, procedures and therapies, outcomes, and comorbidities were recorded. The American Society of Anaesthesiologists’ (ASA) physical status classification and the Charlson Comorbidity Index have been used as measures of physical status [9,10]. A high-dose intravenous infusion of proton pump inhibitors was suggested as per European Guidelines [11] for 72 h for all patients following endoscopic hemostatic treatment of the high-risk stigmata bleeding source. Following initial endoscopic control of bleeding, further endoscopic therapy was performed using local management protocols. Possible comorbidities and the variables useful for establishing the Charlson Comorbidity Index and Glasgow–Blatchford score were searched [10,12].

The following data were recorded from each included hospital: complexity degree (teaching, acute care, or local hospital), type (public, private, or mixed), volume of activity (small, medium, or large), presence of multispecialty settings (cardiology, intensive care, neurology, oncology, gastroenterology, nephrology/dialysis, surgery, geriatrics, pneumology, hematology, oncology units, or organ transplant center), and availability of endoscopy service on call.

Upper endoscopy was performed on all patients with hematemesis. Those with blood in the gastric cavity, irrespective of the detection of the bleeding lesion, were considered patients with upper gastrointestinal bleeding (UGIB) and were subsequently included in this study. Those with melena showing no lesion in the upper digestive tract or no hematic residue in the stomach were considered patients without UGIB. Data on the outcomes, length of hospitalization, stay in the intensive care unit, need for surgery or interventional radiology, further bleeding, and death were collected.

### 2.1. Definitions

AUGIB was characterized by the presence of ongoing overt upper gastrointestinal hemorrhage, with the following symptoms: hematemesis/coffee-ground vomiting, melena (black tarry stool), or a combination of both. All patients were scheduled to undergo upper endoscopy within 24 h from emergency department arrival [11]. During endoscopy, AUGIB was classified as either variceal or nonvariceal; Forrest’s classification was used to categorize the stigmata of nonvariceal bleeding lesions. Further bleeding (persistent or recurrent) was defined according to the criteria by Laine et al. [8]. Need for surgery or interventional radiology, generally following a second failed attempt to perform endoscopic treatment, was decided by each hospital. Mortality from any cause was defined as bleeding-related when it occurred within 30 and 42 days in patients with nonvariceal and variceal bleeding, respectively [13,14].

The time before endoscopy was divided into four time intervals, ≤6, 6–12, 12–24, or ≥24 h, starting from the time of emergency department access according to the ESGE guidelines. Subsequently, the four intervals were correlated with the BleSev score [11].

### 2.2. Bleeding Severity Analysis

Previous studies defined BleSev on the basis of presenting symptoms (hemodynamic shock, hematemesis, and melena) and blood test results (Hb and blood urea nitrogen concentrations and coagulopathy) [5,6,7,8]; in the present study, we also considered mental status, which was defined by the verbal response in the Glasgow Coma Scale as oriented, confused, intelligible single words, incomprehensible sounds, or no audible response [15].

Based on the data reported in the literature, we evaluated the role of the following 12 clinical factors that may be involved in BleSev: demographic (age and gender), hemodynamic (systolic and diastolic blood pressure and heart rate), biochemical (serum urea, Hb, and creatinine levels), and clinical factors (hematemesis, melena, syncope, and mental status at presentation). As a surrogate estimation of BleSev, we considered the worst outcomes patients could be exposed to, such as the need for surgery, interventional radiology, and death; these three outcomes were included in the composite endpoint. As we considered the Hb value as a prerequisite for transfusion, we did not include the “need for transfusion” [16,17]. Moreover, as the most significant prognostic factor for recurrent/persistent bleeding is the type of endoscopic stigmata, which is not available before endoscopy, we excluded further bleeding.

The factors included in the BleSev measure were collected before endoscopy. The independent predictive factors for defining BleSev were identified by univariate and multivariate logistic regression analyses using a backward process.

### 2.3. Statistical Analysis

Sample size calculation for multiple logistic regression was based on the study by Ogundimu et al. [18] and Peduzzi et al. [19] assuming the inclusion of no more than 12 covariates in the final regression and at least 20 events per variable to eliminate bias in regression coefficients when several low-prevalence predictors were included. The minimum sample size was 2.667 ([*n* = 20*C/*p*], where *n* indicates the sample size, with 20 events per variable; C indicates the number of variables expected to be included in the final model; and *p* is the prevalence). The variables of interest were analyzed using descriptive statistics by calculating the median values, proportions, standard deviations, and limits of confidence at 95%, depending on whether the values were continuous, ordinal, or nominal. Univariate analysis was performed by examining the variance, nonparametric test, or test of the proportions according to the typology of variables analyzed. The odds ratios and limits of confidence were calculated at 95%. Variables showing a *p*-value of ≤0.10 at univariate analysis were considered for inclusion in the multiple logistic regression model by a backward process; factors with a *p*-value of <0.05 were subsequently retained in the final model.

### 2.4. Internal Validation

We assessed the model’s predictive accuracy, evaluating whether the model correctly reflected the actual outcome experience in the data (goodness-of-fit). The goodness-of-fit model for derivation of the model’s ability to predict the composite endpoint and 30-day mortality was performed using the Hosmer–Lemeshow test; AUCROC and 95% confidence intervals (CIs) were used for evaluating the discriminative performance for predicting outcomes. The presence of interactions between time to endoscopy, patients’ clinical characteristics, and death, as well as the possible presence of clinical clusters that may describe the risk of death, were evaluated. Patients with missing data were excluded by listwise deletion, and no imputation was performed. All statistical analyses were performed using STATA software version 18.0 (StataCorp LP, College Station, TX, USA). This study was conducted according to the Transparent Reporting of a multivariable prediction model for Individual Prognosis or Diagnosis statement [20].

## 3. Results

Overall, 2525 patients were included (Table 1). The mean age was 68 (±15.8) years, and 1691 patients were men (67.3%). The source of bleeding was nonvariceal in 2079 patients (82.3%), and a shock index of >1 was present in 184 patients (7.7%). Of the patients, 1554 (61.5%) received at least one red blood cell transfusion, 585 (23.2%) of whom were transfused before endoscopy (Table 1). Additionally, of the patients, 27 (1.1%) and 76 (3%) underwent interventional radiology and emergency surgery, respectively; 176 (7%) died due to bleeding, and 253 (10%) developed one of the composite endpoints (Supplementary Materials, Table S1).

Of the 12 factors analyzed at the univariate analysis, only the following 5 factors were retained in the multivariate logistic regression model and were used in BleSev score formation: altered mental status, systolic blood pressure ≤ 100 mmHg, blood urea nitrogen level ≥ 130 mg/dL, hematemesis, and hemoglobin level ≤ 8 g/dL (Supplementary Materials, Table S2). The independent weight of each factor is shown in Table 2, with a maximum point of 10. In the absence of any factor, the death risk was 1%; in the presence of all factors, the death risk was 45.5% (Supplementary Materials, Table S3). An altered mental status was the most significant factor involved in BleSev.

Patients with at least one factor more frequently had a red blood cell transfusion before endoscopy; were older than those without any risk factors; had more advanced comorbidities, cirrhosis, and neoplasia; and had more frequent occurrences of rebleeding and death (Supplementary Materials, Table S4).

Death risk linearly increased with the increase in the BleSev score. We identified the following three risk classes: low (1–2 points), intermediate (3–4 points), and high (≥5 points) (Table 3). Patients without risk factors had a 1% death rate, whereas those in the high-risk class had 21%. The frequency of need for transfusions, need for therapeutic endoscopy, rebleeding, need for surgery or radiological treatment, and length of hospital stay increased with increasing risk classes (Table 4).

**Figure 1:**
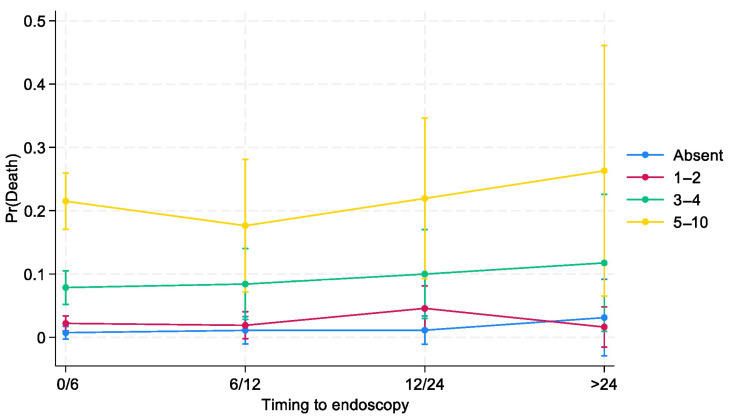
Effects of interactions between time to endoscopy and BleSev on death risk by time to endoscopy.

**Figure 2:**
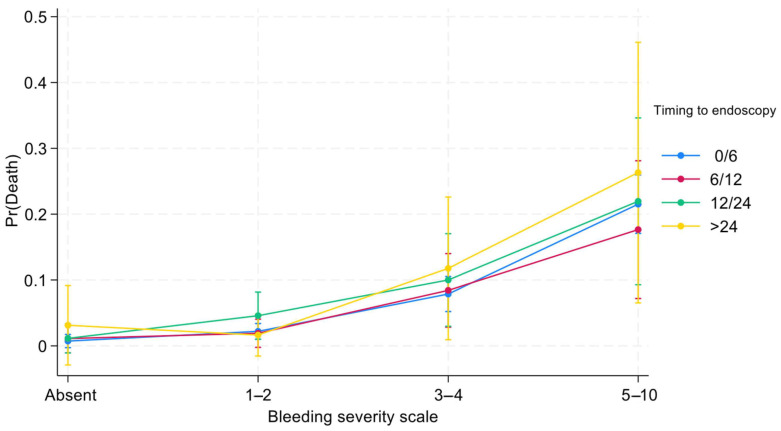
Effects of interactions between time to endoscopy and BleSev on death risk by BleSev scale.

All undesirable clinical outcomes are more frequent in classes with higher bleeding risks, as shown in Figure 1 and Figure 2. For internal validation, we noted no significant difference between observed and predicted frequencies for both composite endpoint (Hosmer–Lemeshow chi2 = 7.80; *p* = 0.55) and death (Hosmer–Lemeshow chi2 = 13.80; *p* = 0.13). The BleSev score linearly predicted the composite endpoint and mortality risk; for the mortality risk, the AUC performance of the scale was 0.79 (95% CI, 0.77–0.81); however, for the composite endpoint, it was 0.73 (95% CI, 0.70–0.76).

### Bleeding Severity and Time to Endoscopy

Three-quarters (75%) of patients with higher (≥5 points) BleSev scores underwent endoscopy within 6 h from hospital admission compared with the 57% of those without any risk factors (Table 4). Overall, the greater the BleSev score was, the shorter the time frame before endoscopy. Using multiple logistic regression analysis, we evaluated the interaction between time to endoscopy and BleSev. The mortality risk increased as the risk class increased, and it was independent from the time to endoscopy (Figure 1) except in the higher risk class (≥5 points), where the mortality rate had a deflection in the 6–12 h time-to-endoscopy group (Supplementary Materials, Figures S1 and S2). Among patients with no BleSev risk factors, mortality risk was approximately 1%, regardless of the time to endoscopy. Within the same risk class, the time to endoscopy did not influence the mortality risk, whereas it showed marked influence in the BleSev 0 and 1–2 groups (Figure 2).

## 4. Discussion

The severity of an UGIB event is frequently defined on the basis of the presenting symptoms and blood test results, along with endoscopic data, including the source of bleeding and endoscopic findings. However, not all of these factors are immediately available and measurable at admission when a prompt and complete evaluation of the clinical severity of a patient with bleeding is crucial to optimize clinical management and, in turn, decrease the risk of death [5,6,7,8].

Here, we focused on identifying independent risk factors involved in the severity of a bleeding event and their weight to define the probability of a single patient dying due to BleSev-related factors.

Of twelve candidate predictors of the bleeding entity, we identified the following five pre-endoscopic independent factors: altered mental status, systolic blood pressure, blood urea nitrogen level, hematemesis, and hemoglobin level (Table 2). Each of these risk factors has a specific weight, depicting a score from 0 to 10 points, with three classes of death risk as follows: low (1–2 points), intermediate (3–4 points), and high (≥5 points). For 0 points, the death risk is 1%; however, in the presence as many as five factors, the death risk increases to 45.5% (Supplementary Materials, Figure S2). Regarding the weight on mortality risk, altered mental status and hypotension (e.g., systolic blood pressure < 100 mmHg) were the most relevant factors contributing to BleSev. Furthermore, the BleSev score showed a good correlation with major outcomes, including the need for red blood cell transfusions, rebleeding rates, length of hospital stays, and need for therapeutic endoscopy (Table 4).

In our cohort, the time to endoscopy did not influence the death risk by the BleSev score in low and intermediate risk classes. As shown in Figure 1, the mortality rate remains constant in patients without any risk factors and in the low- and intermediate-risk groups; in contrast, among those in the high-risk group, the mortality rate decreased in those who underwent endoscopy at 6–12 h from ER arrival and increased when endoscopy was performed after 24 h (Figure 2). This finding suggests that although most patients’ outcomes are not influenced by the time to endoscopy, those in the highest risk category can benefit from more urgent endoscopic interventions. The time to endoscopy remains a critical point among endoscopists who should manage patients with bleeding. Defining the time to endoscopy is fundamental not only for organizational purposes (e.g., defining the need for an on-call 24/7 endoscopy service or a hub-and-spoke network to correctly manage patients with bleeding) but also for clinical and economic aspects (e.g., length of hospital stay, need for high-complexity clinics, and intensive care unit admission). International guidelines have recommended that endoscopy should be performed within 24 h from admission and that outpatient management is a safe option for patients with a Blatchford score of ≤1 or with the support of artificial intelligence systems [21,22]. A previous study from the GISED Study Group demonstrated that in hospitalized patients, the endoscopy time should be tailored to patients’ physical status according to the ASA score, with particular attention to patients with ASA IV scores for whom a “not too early, not too late” rule (12–24 h from admission) was the ideal time frame to minimize mortality risk [23]. Lau et al. demonstrated that in patients with a Glasgow–Blatchford (GB) score of ≥12 (e.g., at a high risk of death), the mortality rate was 8.9% if the endoscopy was performed within 6 h following gastroenterologist consultation and 6.6% if the endoscopy was performed between 6 and 24 h (*p* < 0.34) 7. However, when we investigate these studies, we should keep in mind that a GB score of >12 characterizes only a minority (10%) of patients with bleeding, that the score was validated to discriminate patients who should be admitted versus those who can safely be managed as outpatients, and that its use in different clinical scenario arises from the lack of a dedicated tool for this purpose [24]. Other scores, including the shock index as a surrogate of BleSev, should be abandoned as they performed poorly compared with other pre-endoscopic scores [25]. Moreover, published papers on endoscopic timing provided heterogeneous results, maybe because of the use of different time frames or samples with different comorbidities, or BleSev was frequently declared using own or nonvalidated instruments [26]. Consequently, the significance of defining BleSev using a dedicated clinical model that could enhance the assessment profile of mortality risk may be of great interest and would be recommended for patients with bleeding as it may help clinicians to understand how different severity degrees require different healthcare choices.

This study had one limitation, particularly its reliance on a database that was established 10 years ago and could potentially not represent the actual scenario. However, in the last 10 years, the epidemiology of gastrointestinal bleeding and endoscopic management has not substantially changed. In the present study, we referred to the use of pre-endoscopic factors; therefore, we can still consider our results applicable in clinical practice.

### Perspective

Having an accepted and documented definition of BleSev is highly significant for characterizing the severity of a bleeding event in individual patients. From a clinical and management perspective, BleSev is a measure of the propensity to death due to blood loss; identifying the comorbidity status and performing medical and endoscopic treatments are factors that should be considered for decreasing death risks.

A more tailored and personalized clinical approach may be essential in reducing this risk. Our study demonstrates that the timing of endoscopy significantly impacts mortality risk in a specific subset of patients. Therefore, clinicians must accurately assess the severity of bleeding to prevent overgeneralization. From a scientific perspective, this clinical model could be valuable for studies analyzing upper gastrointestinal bleeding (UGIB). Establishing a clear definition of bleeding severity is crucial for ensuring greater consistency in future research. In particular, it would be interesting to compare this model with existing pre-endoscopic scoring systems for patients with UGIB. Additionally, future studies could assess the model’s predictive value for early versus late mortality and the need for admission to an intensive care unit.

BleSev, clinical characteristics of the patients, and therapy are the three “cornerstones” that should be explicitly declared in scientific works to make the outcomes of patients with acute digestive hemorrhage comparable.

## 5. Conclusions

BleSev influenced the death risk, which increased as the number of risk factors present at the same time increased. Three progressive classes of severity were identified, including low, intermediate, and high risk. Of the patients with BleSev, 57.8% were mild or had no other risk factors present; in this large group of patients, the risk of death was <2%, and the time to endoscopy did not influence mortality. Therefore, although most patients’ outcomes are not influenced by the time to endoscopy, those in the highest risk category may benefit from more urgent endoscopic interventions. The findings underscore the significance of promptly assessing BleSev to guide treatment decisions and improve patient outcomes.

## Figures and Tables

**Table 1:** Baseline features of the cohort at the emergency department.

	Number of Patients out of 2525
Age, mean (±SD)	68 (±15.8)
Male, *n* (%)	1691 (67.3%)
Outpatients, *n* (%)	2142 (84.8%)
Cardiac frequency, bpm mean (±SD)	89.3 (±16.5)
Mean systolic blood pressure, mean (±SD))	115.6 (±22.6)
Mean diastolic blood pressure, mean (±SD)	67.4 (±12.9)
Hemoglobin levels, g/dL (IQR)	8.8 (7.4–10.7)
Blood urea nitrogen, mg/dL (IQR)	58.0 (38–92)
Clinical presentation	
Syncope, *n* (%)	256 (10.1%)
Altered mental status, *n* (%)	420 (16.6%)
Hematemesis, *n* (%)	1127 (44.6%)
Melena, *n* (%)	1660 (65.7%)
Comorbidities	
ASA score	
I	648 (25.7%)
II	856 (33.9%)
III	855 (33.9%)
IV	166 (6.6%)
Renal failure	326 (12.9%)
Chronic coronary artery disease, *n* (%)	552 (21.9%)
Chronic obstructive pulmonary disease, *n* (%)	284 (11.2%)
Neoplasia, *n* (%)	408 (16.2%)
Cirrhosis, *n* (%)	537 (21.3%)
CHILD score, mean (IQR)	8 (6–10)
Number of patients receiving RBC transfusion before endoscopy, *n* (%)	585 (23.2%)
Nonvariceal source of bleeding, *n* (%)	2079 (82.3%)

RBC: red blood cell.

**Table 2:** Weighted risk factors for bleeding severity.

	Weight
Altered mental status, yes	3
Systolic blood pressure ≤ 100 mmHg	2
Blood urea nitrogen level ≥ 130 mg/dL	2
Hemoglobin level ≤ 8 g/dL	2
Hematemesis, yes	1
Total	10

**Table 3:** Death risk by classes of bleeding severity value.

Death by Any Cause			
	Odds Ratio	*p*	(95% CI)
No risk factors (reference)	1		
1–2	2.41	0.077	0.91–6.37
3–4	9.25	0.000	3.67–23.36
≥5	26.29	0.000	10.59–65.27

**Table 4:** Relationship between major clinical outcomes, endoscopic stigmata, therapy, and bleeding severity scale.

		Bleeding Severity Scale				
	Total	0	1–2	3–4	≥5	*p*-Value
	*n* = 2525	*n* = 498	*n* = 965	*n* = 606	*n* = 456	
Time lapse from the ED to endoscopy (h)						<0.001
0–6, *n* (%) 6–12, *n* (%) 12–24, *n* (%) >24, *n* (%)	1589 (64.6) 393 (16.0) 330 (13.4) 146 (5.9)	275 (56.7) 90 (18.6) 88 (18.1) 32 (6.6)	590 (62.8) 157 (16.7) 131 (14) 61 (6.5)	394 (66.4) 95 (16.0) 70 (11.8) 34 (5.7)	330 (72.3) 51 (11.6) 41 (9.3) 19 (4.3)	
High-risk stigmata at index endoscopy, *n* (%)	771 (30.5)	158 (31.7)	276 (28.6)	172 (28.4)	165 (36.2)	0.017
Need for therapeutic endoscopy, *n* (%)	1477 (58.5)	230 (46.2)	543 (56.3)	387 (63.9)	317 (69.5)	<0.001
Transfusion(s), *n* (%)	1554 (61.5)	170 (34.1)	558 (57.8)	423 (69.8)	403 (88.4)	<0.001
Transfusion number, (IQR)	2 (0–3)	0 (0–2)	1 (0–3)	2 (0–3)	3 (2–4)	<0.001
Rebleeding, n (%)	157 (6.2)	13 (2.6)	33 (3.4)	45 (7.4)	66 (14.5)	<0.001
Need for surgery or interventional radiology, *n* (%)	98 (3.9)	15 (3)	29 (3)	27 (4.5)	27 (5.9)	0.035
Death by any cause, *n* (%)	176 (7)	5 (1)	23 (2.4)	52 (8.6)	96 (21.1)	<0.001
Length of stay, day(s) mean (± SD)	9.7 (± 8.8)	7.9 (± 7.1)	9.2 (± 8.9)	10 (± 8.3)	12.3 (± 10.3)	<0.001

Missing data for time lapse for 67 patients (2.7%). ED: emergency department; SD: standard deviation; IQR: interquartile range.

## Data Availability

The data presented in this study are available on request from the corresponding author due to privacy and ethical reasons.

## Supplementary Materials

The supporting information can be downloaded at https://www.mdpi.com/article/10.3390/jcm14051643/s1. Figure S1. Relationship between bleeding severity scale and the composite endpoint; Figure S2. Relationship between bleeding severity scale and death risk; Table S1. Clinical outcomes; Table S2. Independent risk factors for bleeding severity and relative Odds Ratio by multivariate logistic regression; Table S3. Death frequency by bleeding severity scale; Table S4. Clinical data according to the bleeding severity;

## Abbreviations

BleSev	bleeding severity
ASA score	American Society of Anesthesiologists physical status classification system
GB score	Glasgow–Blatchford score
Hb	hemoglobin
PPI	proton pump inhibitor
RBC	red blood cell
ED	emergency department
NSAIDs	nonsteroidal anti-inflammatory drugs
Bpm	beat per minute
SD	standard deviation
UGIB	upper gastrointestinal bleeding
95% CI	95% confidence interval
