# Supplementary material for: Defining and Evaluating the Impact of Bleeding Severity on Time to Endoscopy and Mortality Risk: A Prospective Multicenter Cohort Study
Source: J Clin Med. 2025 Feb 28;14(5):1643. doi: 10.3390/jcm14051643 (PMC11899910; doi:10.3390/jcm14051643)
Supplement: Supplementary file 1 [file jcm-14-01643-s001.zip › jcm-3487823-Supplementary.pdf]

Table S1. Clinical outcomes.

**Need for red blood cell transfusion, n (%)**	**1.554 (61.5%)**
**Rebleeding, n (%)**	157 (6.2%)
**Need for interventional radiology, n (%)**	27 (1.1%)
**Need for surgery, n (%)**	76 (3%)
**Death by any cause, n (%)**	176 (7%)

Table S2. Independent risk factors for bleeding severity and relative Odds Ratio by multivariate logistic regression.

**Bleeding Severity**	**Odds ratio**	***p***	**(95% C.I.)**
**Hematemesis**	1.48	0.006	1.11-1.95
**Haemoglobin ≤ 8gr/dl**	1.62	0.001	1.23-2.14
**Blood Ureic Nitrogen ≥130 mg/dl**	1.79	0.001	1.28-2.52
**Systolic blood pressure ≤100mmHg**	2.27	0.000	1.72-3.00
**Altered Mental Status**	3.37	0.000	2.52-4.49

Table S3. Death frequency by bleeding severity scale.

	**Death rate**		
**Bleeding Severity scale**	No (%)	Yes Nr (%)	Total
**0**	493 (99)	5 (1)	498
**1**	429 (97.06)	13 (2.94)	442
**2**	513 (98.09)	10 (1.91)	523
**3**	329 (91.64)	30 (8.36)	359
**4**	225 (91.09)	22 (8.91)	247
**5**	173 (85.22)	30 (14.78)	203
**6**	74 (80.43)	18 (19.57)	92
**7**	54 (76.06)	17 (23.94)	71
**8**	46 (66.67)	23 (33.33)	69
**9**	7 (70.00)	3 (30)	10
**10**	5 (54.55)	6 (45.45)	11
**Total**	2.349	176	2.525

Pearson chi^2(10) = 241.9261 Pr = 0.000

Table S4. Clinical data according to the bleeding severity.

	**Total**	**No risk factors**	**At least one risk factor**	**p-value**
	N=2.525	N=498	N=2.027	
Age, mean (SD)	68.0 (±15.8)	66.4 (±16.1)	68.4 (±15.7)	0.014
In-patients bleeding, n (%)	1.691 (67.3)	354 (71.5)	1.337 (66.3)	0.027
In hospital bleeding	380 (15.5)	77 (15.9)	303 (15.4)	0.79

Pre-endoscopic PPI use, n (%)	1.815 (71.9)	344 (69.1)	1.471 (72.6)	0.12
ASA score				
I	648 (25.7)	182 (36.5)	466 (23)	
II	856 (33.9)	130 (26.1)	726 (35.8)	
III	855 (33.9)	173 (34.7)	682 (33.6)	<0.001
IV	166 (6.6)	13 (2.6)	153 (7.5)	
Pre-existing comorbidities, n (%)				
Renal failure	326 (12.9)	44 (8.8)	282 (13.9)	0.002
Chronic coronary artery disease	552 (21.9)	124 (24.9)	428 (21.1)	0.067
Chronic obstructive pulmonary disease	284 (11.2)	41 (8.2)	243 (12)	0.017
Any neoplasia	408 (16.2)	48 (9.6)	360 (17.8)	<0.001
Cirrhosis	537 (21.3)	38 (7.6)	499 (24.6)	<0.001
Antiplatelets agents, n (%)	602 (23.8)	135 (27.1)	467 (23)	0.056
Non-vitamin K oral anticoagulants, n (%)	153 (6.1)	30 (6)	123 (6.1)	0.97
NSAIDs, n (%)	518 (20.5)	117 (23.5)	401 (19.8)	0.066
Anticoagulant agents, n (%)	243 (9.6)	59 (11.8)	184 (9.1)	0.060
Cardiac frequency, bpm (± SD)	89.3 (±16.5)	85.2 (±15.2)	90.3 (±16.7)	<0.001
Mean systolic pressure (± SD)	115.6 (±22.6)	127 (±17.1)	112.8 (±22.9)	<0.001
Hemoglobin levels, gr/dl (IQR)	8.8 (7.4-10.7)	10.1 (8.9-11.9)	8.4 (7-10.3)	<0.001
Non variceal source of bleeding, n (%)	2.079 (82.3)	471 (94.6)	1.608 (79.3)	<0.001
High risk stigmata, n (%)	771 (30.5)	158 (31.7)	613 (30.2)	0.52
Need for therapeutic endoscopy, n (%)	1.477 (58.5)	230 (46.2)	1.247 (61.5)	<0.001
Number of patents receiving RBC transfusion, n %	1.554 (61.5)	170 (34.1)	1.384 (68.3)	<0.001
RBC transfusion per patient, n (IQR)	2 (0-3)	0 (0-2)	2 (0-3)	<0.001
Lenght of stay, day(s) (± SD)	9.7 (± 8.8)	7.9 (± 7.1)	10.1 (± 9.2)	<0.001
Rebleeding, yes (%)	157 (6.2)	13 (2.6)	144 (7.1)	<0.001
Need for interventional radiology or surgery, yes (%)	98 (3.9)	15 (3)	83 (4.1)	0.26
Death by any causes, yes (%)	176 (7)	5 (1)	171 (8.4)	<0.001

PPI: proton pump inhibitors; NSAIDS: non-steroidal anti-inflammatory drugs; Bpm: beat per minute; RBC: Red blood cell

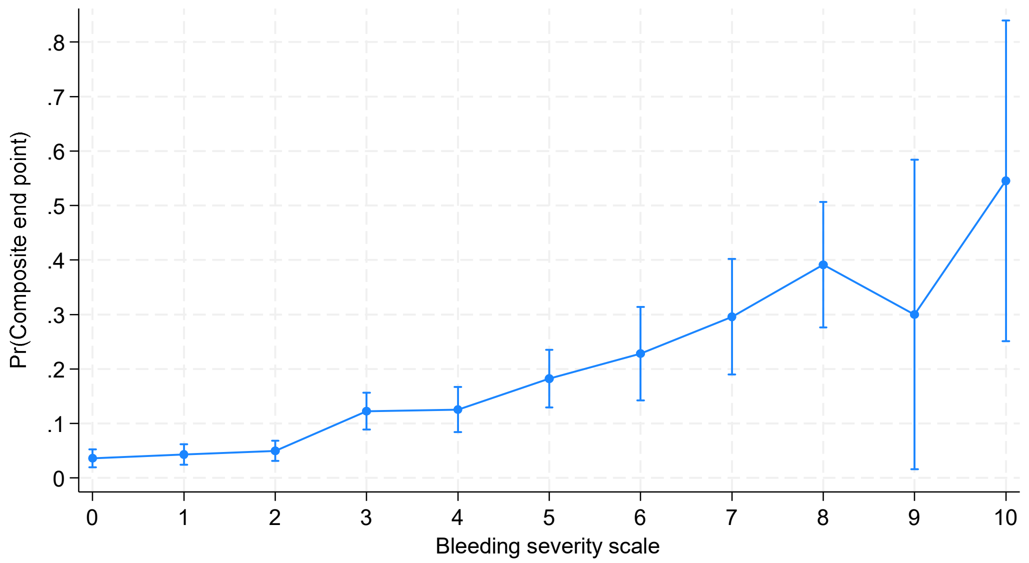

Figure S1. Relationship between bleeding severity scale and the composite endpoint

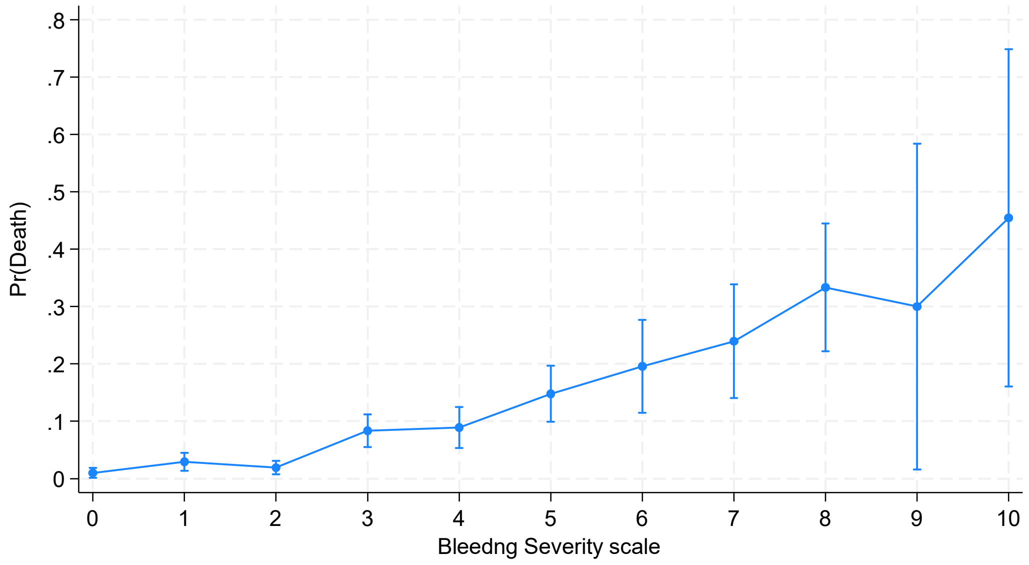

Figure S2. Relationship between bleeding severity scale and death risk
